# Supplementary material for: Symbiotic and Asymbiotic Germination of Dendrobium officinale (Orchidaceae) Respond Differently to Exogenous Gibberellins
Source: Int J Mol Sci. 2020 Aug 25;21(17):6104. doi: 10.3390/ijms21176104 (PMC7503528; doi:10.3390/ijms21176104)
Supplement: Supplementary file 1 [file ijms-21-06104-s001.pdf]

Supplementary Table S1. Primer sequences used in qRT-PCR amplification. All primers were designed using Primer 3.0 software and synthesized by Genewiz Company (China).

**Gene ID**	**Gene name**	**Forward primer sequence（5'-3'）**	**Reverse primer sequence（5'-3'）**
-	*EF-1α*	TCAGGCTGACTGTGCTGTCCT	GTGGTGGCGTCCATCTTGTT
Dendrobium_GLEAN_10138923	*DoKO*	CTCTAGTCACCAAGTTCTCATC	CCATAGTCGCATGTGGATAC
Dendrobium_GLEAN_10043501	*DoGA3ox*	TCCGACGAACTCTACCAAGGTTACA	AACTCCATACTCAGACAGCCGAACT
Dendrobium_GLEAN_10025219	*DoGA2ox*	GAAGCTTTCATTGCATGGTC	GTTGTGTCTCTCTCCTTCATC
Dendrobium_GLEAN_10048964	*DoGA20ox*	GGAAGCCACCGACAATTTA	GATGCGATGGTTCACTACTT
Dendrobium_GLEAN_10024051	DoSCL3	ATGGTGGTGACTGAACAGGAATCG	GCACAGTTGACTCCAAGCAATCG
Dendrobium_GLEAN_10081660	*DoGBF*	GAGGTGACTTTGGAGGATTAC	GATTCGTGTATTGCTGCTTTC
Dendrobium_GLEAN_10040978	*DoIRK*	ATGGCGACTTAGGCTGGACTACA	AGGCAGAGGAGATGGAAGGCTAC
Dendrobium_GLEAN_10062985	*DoSGT*	CCTCCGCCTCTATGAATCT	CGGCGGAAAGGTGATAATAG
Dendrobium_GLEAN_10070249	*DoNCED*	GAGATGCCGTTAAGGTACAG	CGCCTCGTATGCGTTTAT
Dendrobium_GLEAN_10012601	*DoIPM*	ACTTCCTCTCCTACCATACC	AACACGGGCTCATCATTC
Dendrobium_GLEAN_10075175	*DoSAUR71*	ACGTGCCAGTCTTTGTAG	CTCTGCTCGTAACCATACTC
PEQU_11738-D2	*DoNSP2*	GCGTCCACATCGTTGATTA	GAGTCTTCTTCCTGTCTCTTTG
Dendrobium_GLEAN_10030409	*DoNSP2*	CGACCTCCCTTCTTTCATTC	CTCTGAGCCGAACCAATATC
Dendrobium_GLEAN_10016982	*DoCDPK 26*	TGTGCGTCGTGAGATCCAGATAATG	CCGCCTTCCTCTCGCTATAATGC
Dendrobium_GLEAN_10048053	*DoCML 19*	GTCGTTGGAGAATACGAAGAG	TCCGAATCCAAAGACTTAACC
Dendrobium_GLEAN_10042237	*DoHAL*	CCAACTATCTCTCCGGCTAT	TCCACGCATCCATCAAATC
Dendrobium_GLEAN_10113668	*DoGGLU*	CCTTTCTCCCAAGACGTAAAT	CCGGTGAGGAAACCAATAAT
Dendrobium_GLEAN_10033071	*DoGLU*	GGCTAATGCACAGACCTATC	CTGCTTGTCGGGAGTAAAC
Dendrobium_GLEAN_10044017	*DoPRCP*	GAGACCAGTGAGAGTTGTTATC	CTCCACTTCGGATTGTCATAC
Dendrobium_GLEAN_10125587	*DoSWEET14*	TGGGTTTCCTGTTTGGATTAG	GGCTTGACTGCTATGTTCTT
Dendrobium_GLEAN_10098792	*DoCDR1*	GAATCCCAGTGCGAGTATAAC	GGAGGCAGTAGGAGAACTTA
